# Pathology of the Supra-Renal Capsules

**Published:** 1858-08

**Authors:** 


### EDITORIAL DEPARTMENT.

*Pathology of the Supra-Renal Capsules.*—The great interest which the paper of Dr. Addison excited in the profession, and the light which it apparently threw upon an obscure disease, (bronzed skin,) and the function of the supra-renal capsules, induced us to make an analysis of an admirable paper by Dr. Harley, which appeared in the British and Foreign Medico-Chirurgical Review. This analysis appeared in our March number, and we then promised to conclude it, when the second part of Dr. Harley's paper appeared. The first part of this paper was devoted to experimental researches on the function of the supra-renal capsules, and the effect, upon the lower animals, of their extirpation. The second part which appeared in the April number of the British and Foreign, discusses the pathology of these bodies.

Before the publication of Dr. Addison's monograph, little was known or thought about the functions of the supra-renal capsules; they were thought to be organs which exercised their function most actively, if not entirely, during fœtal life; and as a natural consequence of the obscurity of their physiology, almost nothing was known of their pathology. Dr. Addison, however, seemed to establish an invariable connection between a discoloration of the integument, known as bronzed skin, and degeneration of these organs; but subsequent investigations, and especially the observations of Harley, have placed this matter again *sub judice.*

In the discussion of this question, there are two important points to be ascertained: Firstly, "Is supra-renal capsular disease invariably accompanied by bronzed skin? and, secondly, Is bronzed skin always associated with disease of these organs?"

Every one will see, that in order to establish an undoubted connection between disease of these bodies and bronzed skin, both these questions must be answered in the affirmative; and at the time when Addison published his monograph, there were a number of cases which seemed to establish these facts, and some which tended to disprove them. Now, however, the case is different; and in reply to the first question, Dr. Harley cites twelve examples of extensive disease of the supra-renal capsules without discoloration of the skin:

"Professor Friedriech relates a case of extensive amyloid degeneration of the supra-renal capsules, without a trace of bronzing of the skin. He says that the organs were of normal size, but unusually hard. On being cut into, the brown cortical pigment zone was found entirely absent. On microscopical examination, this part of the organ was seen to be full of fat granules. The medullary substance was even in a still more advanced stage of degeneration; the greater part of the cells being transformed into homogeneous glittering masses — a fact, as the author remarks, of particular importance in connection with the absence of any discoloration of the skin.

"Professor Friedriech in the same paper, mentions still another case, in which the supra-renal capsules were found of twice their hormal size, and extensively affected with amyloid degeneration. The microscopical characters were similar to those mentioned as occurring in the other specimens, and yet there was no discoloration of the skin."

These cases we have copied in full, as being perfect and complete in every particular; the capsule being examined with the scalpel and microscope, and by one whose authority cannot be disputed. Dr. Harley mentions ten other cases of a similar character; one communicated to the Bath and Bristol Association, by Dr. Davis; several by Prof. Vinchow; and cases reported by Drs. Peacock, Bristowe, John Ogle, Rokitanski, and Prof. Martini, of Naples. Dr. Harley mentions a case which he examined himself, the patient having died at the Middlesex Hospital and the capsules being forwarded to him by Dr. Van der Byl.

"The capsules at first sight appeared healthy. On being cut into, however, the sinuses of the medullary part were found all united into one, so as to form a tolerably large cavity, which contained a dark grumous looking fluid. On examining this fluid with the microscope, it was seen to contain a number of blood corpuscles, pigment granules, oil globules, and broken up nucleated cells,—most probably part of the *debris* from the medullary substance, which was found no longer to possess its normal histological structure. The cortical portion of the capsules was of a dingy yellow color, soft, and exceedingly friable; so much so, that on attempting to make a thin section of it with a knife, it crumbled away before the cutting edge. On placing a small portion under the microscope, there was neither the slightest trace of loculi or fibrous matrix visible; nothing but a confused mass of cells, granules, and fatty matter could be detected. In fact, it was almost entirely composed of fat globules and granules."

From notes of this case which Dr. Harley obtained from Dr. Van der Byl, it was shown that the patient was of an extremely scrofulous habit, and presented fatty degeneration of the heart, liver and spleen; there was no discoloration of the skin.

One cannot fail to agree with Dr. Harley, after a perusal of the preceding cases, that the question, "Is disease of the supra-renal capsules invariably accompanied by bronzed skin?" is emphatically answered in the negative. In pursuing the subject, then, the second question presents itself, *i. e.*, "Is bronzed skin always associated with disease of the supra-renal capsules?"

We have seen, by one of the preceding cases, that in the investigations bearing upon this point, the organs must be carefully examined, and examined microscopically; as in the case which was examined by Dr. Harley himself, in which there was extensive disease. "The capsules at first sight appeared healthy," and this is the case in many other instances which have been quoted. In examining the cases of bronzed skin which are mentioned in this article, we find that they are generally accompanied by headache, nausea, constipation, either constant, or alternating with diarrhœa, and frequently, great derangement of the digestive system; it is by no means uncommon for the patients to be affected with phthisis or some other serious disease. This is a general view of the functional derangements which accompany the disease, which is sometimes named, after its discoverer, Addison's disease. There are other cases of discoloration of portions of the integument which occur without general derangement, and which will be referred to hereafter. We quote a case of bronzed skin without disease of the capsules, reported by Dr. Fricke:

"Dr. Fricke has published a case of an Irishman, aged twenty-five, who, in the latter part of 1856, complained of debility, nausea, headache, and constipation. A month afterwards, this man was attacked with jaundice, from which he gradually recovered. Last January (1857) it was noticed that he had a bronzing of the skin. It gradually became more decided. On the 29th of April last, the man died, and on post-mortem examination his supra-renal capsules were found of the normal size, hue, and consistence, presenting no alteration whatever. There was very marked cirrhosis of the liver."

M. Puech also communicated an analogous case to the French Academy, April 6th, 1857. It was a well marked instance of bronzed skin, which had existed for nearly two years, and on post-mortem examination, no change whatever in the capsules was revealed by the most minute examination. Cases have been reported by Profs. Simpson, Vrichow, Dr. Suton, Dr. Alexander Simpson, Mr. May, and Mr. Hutchinson, of well marked bronzed skin and *no disease of the supra-renal capsules.*

The second question then has been satisfactorily answered. Bronzed skin is not always associated with supra-renal capsular disease. That bronzing of the skin is not the result of a particular disease, or class of diseases affecting the supra-renal bodies, is abundantly shown by the experience of Dr. Addison himself, who has found it to be associated with cancer, tubercle, and a variety of other diseased conditions of the capsule. Bronzing of the skin is not due to a suppression of the function of the supra-renal capsules, as is shown by the cases in which they were absent, or absolutely destroyed, and not followed by discoloration of the skin.

Thus far, Dr. Harley has undoubted proved that the cause of bronzed skin is not invariably a disease of the supra-renal capsules, and now the question naturally follows, WHAT IS THE CAUSE OF BRONZED SKIN?

To this question it is as yet difficult to reply. We shall not follow closely through the remainder of the article. It is occupied by a discussion of the tints of complexion which are peculiar to different races of men, which are changed by the influence of the sun's rays, which we know to be a powerful stimulant to the formation of pigment, and the varied colors of some animals, as the chamelion, the different colors of the fur of animals and the plumage of birds at different seasons of the year. All these are extremely curious and interesting, and in the latter instances difficult to explain, but they throw no light upon the subject under consideration. There are, also, very curious cases on record, where extensive discoloration of the skin has attended gestation, and disappeared some months after delivery. Some cases of discolored skin are classed among the cutaneous diseases, as the melanopathia syphilitica, chloasma, or maculæ hepaticæ. It is sufficiently well known "that partial or even general discoloration of the skin occurs under such a variety of circumstances that it can scarcely be regarded as depending upon any one particular form of disease."

Dr. Harley has examined with the microscope, specimens of bronzed skin taken from a patient whose supra-renal capsules were diseased, and found that it presented the same appearance as the skin of the negro. He also examined bronzed skin taken from a patient with healthy supra-renal capsules with the same result. He is unable to detect any difference between the skin of a sunburnt or freckled person, or that of a patient affected with Addison's disease, except in as far as the quantity of the pigment is concerned.

From the facts of which we have given a sketch in the preceding pages, the author draws the following conclusions:

"Firstly, That bronzed skin may exist without the supra-renal capsules being diseased. Secondly, That complete degeneration or total absence of the supra-renal capsules may occur without any bronzing of the skin. Thirdly, That bronzed skin may be associated with a variety of different morbid conditions of the system, among which a prominent one is disease of the supra-renal capsules. Fourthly, That bronzed skin may be present without any derangement of the other functions of the body being observed. The treatment of the affection must consequently be varied accordingly.

"Upon the anatomical and physiological grounds previously stated, I look upon the symptoms of anæmia, languor, debility, feebleness of the heart's action, and irritability of the stomach, not as the result of the suppression of the function of the supra-renal capsules, but rather as being occasioned either by a diseased state of the solar plexus, *per se*, or by an irritation of the ganglionic system of nerves, caused by the close proximity and intimate connection of diseased supra-renal capsules.

"The investigation is still far from being completed, and as I am not wedded to any hypothesis, if new facts are discovered which show the subject in a different light, I shall not hesitate to mould my views accordingly."

We have made the preceding analysis, under the conviction that these papers take the most rational view of the subject, of any that have been published. These views are in direct opposition to those advanced by E. Brown-Séquard and M. Martin-Magron, but we think that most of them are endorsed by MM. Gratiolet and Philipeaux. Séquard has made experiments intended to demonstrate that the supra-renal capsules are essential to life, and he offers as proof, that animals which have been deprived of these bodies by himself, have died in a few hours. In opposition to this, Dr. Harley states that he has removed the supra-renal capsules from white mice, and they have survived for months with good health. In answer to this, Brown-Séquard makes the unfounded assertion, which is only called out by this particular fact, that in albinos, the capsules have no function. But this is of disposed by the fact that colored animals have also survived removal of the supra-renal capsules.

We fear that we must still confess ourselves in the dark as regards the function of the supra-renal capsules, and that the invariable connection which Addison seemed to demonstrate between disease of these organs and discoloration of the skin, cannot be sustained.

---

*Erratum.*—In a report by Dr. Lemon, presented to the Buffalo Medical Association, page 111 of the July number, for "the child remained in this position over *fifteen minutes*, read, "the child remained in this position over *five minutes*."

